# Photovoltaic Performances of Dye-Sensitized Solar Cells Based on Modified Polybutadiene Matrix Electrolytes by Sol-Gel Process

**DOI:** 10.3390/polym14122347

**Published:** 2022-06-09

**Authors:** Mi-Ra Kim, Thanh Chung Pham, Hyun-Seock Yang, Sung Heum Park, Songyi Lee

**Affiliations:** 1 Department of Chemistry, Pukyong National University, Busan 48513, Korea; mrkim2@pknu.ac.kr; 2 Division of Chemical Engineering and Materials Science, Ewha Womans University, Seoul 03760, Korea; ptchung.chem@gmail.com; 3 Department of Physics, Pukyong National University, Busan 48513, Korea; randafine@gmail.com (H.-S.Y.); spark@pknu.ac.kr (S.H.P.); 4 Industry 4.0 Convergence Bionics Engineering, Pukyong National University, Busan 48513, Korea

**Keywords:** polybutadiene, dye-sensitized solar cells (DSSCs), polymer electrolyte, sol-gel process

## Abstract

A new type of polymer matrix electrolyte based on modified polybutadiene (modified PB) was developed for dye-sensitized solar cells (DSSCs) to improve their stability. The modified PB was fabricated by cross-linking the reaction of polybutadiene with siloxane groups as a substitute sol-gel process. A DSSC device using the modified PB matrix electrolyte showed an open-circuit voltage of 0.64 V, a short-circuit current density of 15.00 mA/cm^2^, and a fill factor of 0.58 under photointensity of 100 mW/cm^2^ at AM 1.5, consequently leading to an overall solar energy conversion efficiency of 5.49%. The DSSC device using the modified PB matrix electrolyte improved the conductivity, and the charge transfer ability showed the outstanding stability of the device.

## 1. Introduction

Dye-sensitized solar cells (DSSCs) based on dye-adsorbed nanoporous titanium oxide (TiO_2_) are considered to be promising alternatives to conventional silicon solar cells because of their low production cost, simple structure, and easy production [1,2,3,4,5]. However, the presence of traditional liquid electrolytes in these cells has caused a lot of skepticism regarding sealing problems, photochemical stability, and the solubilization capacity of the redox couple. For this reason, substituting the liquid electrolyte with a solid or gel electrolyte has been considered, as it could offer several advantages compared with traditional wet cells.

In some cases, the electrolyte has been replaced by a hole-transporting organic material [6,7,8,9,10,11]. In other cases, a p-type semiconductor has been deposited on the TiO_2_-dye-sensitizer film [12,13,14,15]. Therefore, hole-conducting polymer has been the subject of study aimed at improving the power conversion efficiency of DSSCs. Hole-conducting polymers are of practical interest due to their hole-accepting capability from dye cation. They can also replace liquid electrolytes for the long-term stability of DSSCs. Several researchers have replaced a liquid electrolyte with an organic polymer incorporating the redox couple [16,17,18,19,20]. The efficiency of these DSSCs (so-called solid-state DSSCs) is generally very low. The main reason for such low performance is the lack of sufficient electricity conduction between the working electrode and the organic polymer incorporating the redox couple.

Another method for solving these problems is using quasi-solid-state electrolytes. Although the efficiency of the DSSCs with quasi-solid-state electrolytes is often lower than that of DSSCs with liquid electrolytes, the quasi-solid electrolytes may become viable alternatives to the liquid electrolytes, owing to their improved stability and better sealing ability [21,22].

There are three typical methods for preparing quasi-solid electrolyte: (i) liquid electrolytes are solidified by organic polymer gelators to form thermoplastic polymer electrolytes or thermosetting polymer electrolytes; (ii) liquid electrolytes are solidified by inorganic gelators, such as SiO_2_ nanoclay powder, to form composite polymer electrolytes; (iii) ionic liquid electrolytes are solidified by organic polymer or inorganic gelators to form quasi-solid ionic liquid electrolytes [23,24]. A sol-gel method using the gelator was an easy and simple process, and can be used to increase viscosity and reduce leakage. The gelators can be added to an organic or inorganic (or both) matrix to fabricate a quasi-solid type, so that an organic-inorganic hybrid material can be prepared. Organic-inorganic hybrid materials by a sol-gel process have been studied in the various fields of optical data storage, nonlinear optical, and holographic applications [25,26,27]. The sol-gel process involving hydrolysis and condensation can form various microstructures as a matrix with high optical quality at a low temperature. The large variety of suitable organic molecules makes it possible to control optical, chemical, and mechanical properties [28]. Linear polymers are often used as organic polymer gelators, including poly(ethylene oxide) (PEO or PEG), poly(acrylonitrile) (PAN), poly(vinyl pyrrolidinone) (PVP), polystyrene (PS), poly(vinyl chloride) (PVC), poly(vinylidene ester) (PVE), poly(vinylidene fluoride) (PVDF), poly(methyl methacrylate) (PMMA), etc. [16,29]. Generally, compared to DSSCs based on liquid electrolytes, DSSCs based on polymer gel electrolytes have lower J_SC_ and higher V_OC_; the former is due to the lower conductivity coming from the lower mobility of the redox couple components, and the latter is due to the suppression of dark current by polymer chains covering the surface of the TiO_2_ electrode [21,30,31]. A TiO_2_ nanofiber electrode obtained from the electrospinning method yielded 4.6% energy conversion efficiency with a gel electrolyte [29]. Polymer electrolyte DSSCs based on polyethylene oxide showed an overall efficiency of 4.2% under direct sunlight [30]. A DSSC with a ternary component polymer-gel electrolyte exhibits the best energy conversion efficiency (7.2% at 1 sun) [31].

Concerning polybutadienes as the linear polymer, the alkoxy silanes are grafted onto the polybutadiene backbone by using hydrosilylation of vinyl double bonds or by the radical addition of mercapto silanes on vinyl double bonds. Compounds like alkoxy silanes in polybutadienes are introduced either to conceive new organic-inorganic materials with specific properties or to modify polymers in order to improve their physical, chemical, or mechanical properties. Modified polybutadienes, with its chemical and mechanical stability, is very suitable for applying to the polymer matrix of quasi-solid electrolytes; moreover, it can expand commercial applicability.

On account of the modified polybutadiene structure containing siloxane groups, the lone pair electrons of the oxygen in the siloxane group affect the formation and stabilization of the redox couples (I^−^/I_3_^−^) in the quasi-solid electrolyte. In quasi-solid electrolytes, the redox couples (I^−^/I_3_^−^) can usually be formed from iodine (I_2_) and iodide anion (I^−^), which is formed by the dissociation of LiI, KI, or quaternary ammonium iodide (NR_4_I). The siloxane groups in the modified polybutadiene are sufficient to stabilize the dissociated cations (Li^+^, K^+^, or NR_4_^+^); thus more iodide anions (I^−^) can be generated to be redox couples. The formed redox couples are an important factor for ionic transport and current flow in the quasi-solid electrolyte of DSSCs.

In this study, we designed a modified polybutadiene (modified PB) structure containing siloxane groups that can improve both the chemical/mechanical stability of polymer matrix and the stability of the redox couples in the quasi-solid electrolytes. The alkoxy silane groups were introduced in polybutadiene (PB) backbones by radical addition reaction, and the polybutadiene containing alkoxy silane groups (PBS-Si) was crosslinked by itself without gelators to fabricate modified PB containing siloxane groups. Modified PB containing siloxane groups should play an excellent role as a polymer matrix inside quasi-solid electrolytes, by stabilizing the redox couples.

Our approach obviated the need for additional gelators with its own sol-gel process, and is therefore a method for crosslinking a linear polymer without a crosslinking agent or a gelator. Furthermore, this approach could expand the application possibilities and improve the performance of DSSCs through the development of new polymers.

## 2. Materials and Methods

### 2.1. Materials and Measurements

Poly(butadiene), azobisisobutylonitrile (AIBN), methyl ethyl ketone (MEK), mercapto propyl triethoxy silane, iodide (I_2_), acetonitrile (AN), propylene carbonate (PC), ethylene carbonate (EC), and tetrabutylammonium iodide (TBAI) were purchased from Sigma–Aldrich Co., Ltd (Seoul, Korea). Cis-di(thiocyanato)-N,N-bis(2,2′-bypyridyl-4.4′-dicarboxylic acid)ruthenium (II) complex (N3 dye), fluorine-doped SnO_2_-layered (FTO) glass (8 ohm/sq), Pt-catalyst T/SP, Ti-Nanoxide T/SP, and 1-propyl-3-methylimidazolium iodide (PMII) were purchased from Solaronix, S.A (Aubonne, Switzerland).

^1^H NMR spectra were measured on a Varian spectrometer (300 MHz, Austin, TE, USA) with CDCl_3_ as d-solvent. UV-Vis spectra were obtained on an Optizen 2120 UV spectrophotometer (Deajeon, Korea). Thermogravimetric analyses (TGA) were performed on a TA Instrument (TGA Q50) thermal analyzer (New Castle, DE, USA) at a heating rate of 10 °C/min in nitrogen (40 mL/min). Morphologies and thicknesses of nanoporous TiO_2_ layers were observed with a Field Emission Scanning Electron Microscope (FE-SEM, TESCAN (MIRA 3 LMH In-Beam Detector), TESCAN Korea, Seoul, Korea). The photovoltaic performance of the DSSC devices were investigated using a solar simulator (PEC-L11, PECCELL, Yokohama, Japan) furnished with a 150 W xenon lamp at AM 1.5. The photointensity was 100 mW/cm^2^. The solar simulator was calibrated to Si reference cell verified. The active area of the DSSC devices was measured with a black mask was 0.40 cm^2^. Electrochemical impedance spectroscopy (EIS) data were measured with an impedance analyzer (Reference 600, GAMRY instruments, Warminster, PA, USA) and recorded over a frequency range of 1~10^6^ Hz at room temperature. The applied bias voltage and ac amplitude were set as the open circuit voltage (V_OC_) of the cell and 50 mV, respectively. EIS data were fitted using a Z-MAN software (WONATECH, Seoul, Korea) and Echem analyst (Garmy, Warminster, PA, USA).

### 2.2. Syntheses of PBS-Si and Modified PB

**Figure 1:**
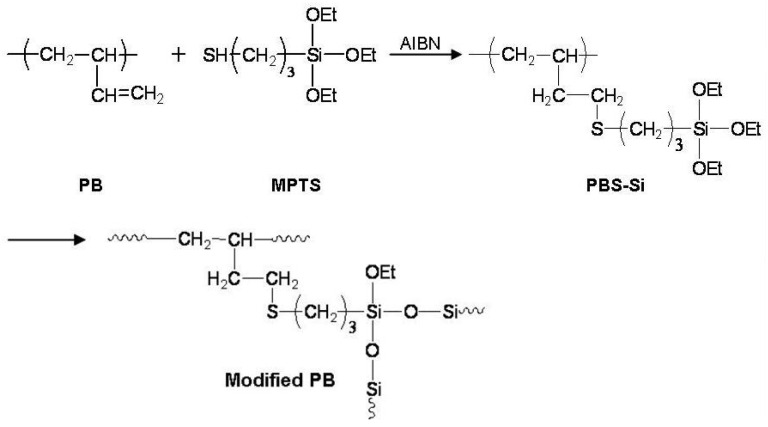
Reaction scheme for PBS-Si and modified PB.

The PBS-Si was synthesized as follows. Firstly, 4.6 × 10^−3^ moles (0.25 g) of 1,2-addition PB and 10% (0.075 g) of AIBN were introduced into a 100 mL round bottom flask equipped with a mechanical stirrer, a switchable inlet for nitrogen, and a vacuum connector. Methyl ethyl ketone (MEK) (14 mL) was added via a syringe. The temperature was raised to 75 °C under nitrogen. After adding 6.9 × 10^−3^ moles (1.65 g) of mercapto propyl triethoxy silane in MEK (6 mL), the reaction mixture was stirred at 87 °C for 48 h under a flow of nitrogen. The reaction was allowed to proceed until consumption of thiol functions was complete. PBS-Si containing triethoxysilane group was obtained as a viscous liquid with a yellow color [32,33] (Figure 1). The modified PB was fabricated by cross-linking reaction with itself for about 72 h. The PBS-Si was analyzed by ^1^H NMR and FT-IR spectroscopy, and compared with those of the initial PB spectra. The ^1^H NMR spectra of PB and PBS-Si are shown in Figures S1 and S2, respectively. The modified PB was analyzed by FT-IR spectroscopy.

Spectra data of PB: ^1^H-NMR (cm^−1^, δ) 5.53–5.33 (m, 1H, -CH=CH_2_), 4.95–4.87 (t, 2H, -CH=CH_2_), 2.12–1.97 (t, 1H, -(CH_2_-CH)-), 1.26–1.17 (m, 2H, -(CH_2_-CH)-). Spectra data of PBS-Si: ^1^H-NMR (cm^−1^, δ) 3.84 (q, 6H, -OCH_2_CH_3_), 2.65–2.18 (m, 4H, -CH_2_-S-CH_2_-), 1.82–1.58 (m, 4H, -CH_2_-CH_2_-S-CH_2_-CH_2_-CH_2_-Si), 1.23 (t, 9H, -OCH_2_CH_3_), 1.10–1.03 (m, 1H, -(CH_2_-CH)-), 1.01 (s, 2H, -(CH_2_-CH)-), 0.74 (t, 2H, -CH_2_-Si-). Spectra data of PB: FT-IR: ν = 3081 (sp^2^ C-H stretch), 2975–2844 (sp^3^ C-H stretch), 1639 (C=H stretch). Spectra data of PBS-Si: FT-IR: ν = 2975–2844 (sp^3^ C-H stretching), 1169–1111 (Si-O-C asym stretching), 956 (Si-O-C sym stretching). Spectra data of modified PB: FT-IR: ν = 2975–2844 (sp^3^ C-H stretching), 1169–1111 (Si-O-C asym stretching), 956 (Si-O-C sym stretching).

### 2.3. Fabrication of DSSC Device

The DSSC devices were prepared using cis-bis(isothiocyanato)bis(2,2’-bipyridyl-4,4’-dicarboxylato)-ruthenium(II) dye (N3 dye, Solaronix Co.) as a photosensitizer, sandwiched with a TiO_2_-deposited and a Pt-coated electrode as two electrodes. The DSSC devices were fabricated according to procedures described previously [34,35].

In order to comparing the photovoltaic performances of the DSSC devices as the electrolyte, three kinds of electrolytes were prepared: (1) Electrolyte. The electrolyte consisted of I_2_, tetrabutylammonium iodide (TBAI), 1-propyl-3-methyl imidazolium iodide (PMImI) as an ionic liquid, and ethylene carbonate (EC)/propylene carbonate (PC) (EC:PC = 4:1 *v*/*v*); (2) PB/electrolyte. The PB solution (200 mg in 0.4 mL of MEK) was coated onto dye-coated TiO_2_ electrode by a spin-coating method (800 rpm, 20 s). The electrolyte prepared at (1) was immediately dropped onto the coated PB film. The coated PB film containing the electrolyte was dried for 2 h at 55 °C to remove the solvent; (3) Modified PB/Electrolyte. The PBS-Si synthesized in Figure 1 was coated onto dye-coated TiO_2_ electrode by a spin-coating method (800 rpm, 20 s). The electrolyte prepared at (1) was immediately dropped onto the coated PBS-Si film. The gelation of PBS-Si was formed by cross-linking reaction with itself during about 72 h without additional gelators, then the modified PB, containing the electrolyte prepared at (1), was fabricated.

**Figure 2:**
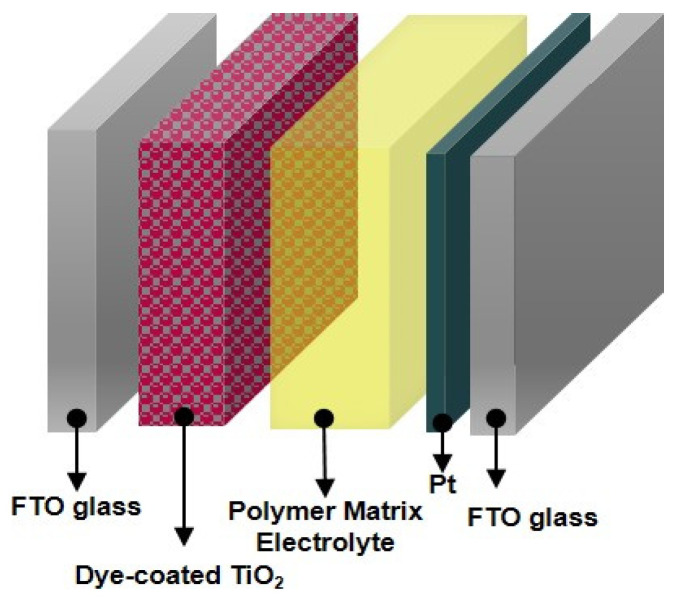
Schematic diagram for DSSC device using polymer matrix electrolyte based on PB or modified PB.

The edges of the devices were sealed with Amosil 4 to protect them against corrosion during a long-term stability test. The DSSC devices were stored in darkness at room temperature. A schematic of a DSSC device using a polymer matrix electrolyte based on PB or modified PB is shown in Figure 2.

## 3. Results and Discussion

In general, a DSSC consists of a dye-coated semiconductor electrode and a counter electrode arranged in a sandwich configuration with its inter-electrode space filled with an electrolyte containing a redox mediator (I_3_^−^/3I^−^). Photoexcitation of metal-to-ligand charge transfer of the adsorbed sensitizer leads to injection of electrons into the conduction band of the semiconductor-titanium oxide. The oxidized dye is subsequently reduced by electron donation from an electrolyte containing an iodide/triiodide redox mediator. The injected electron flows through the semiconductor network to arrive at the back contact. It then flows through the external load to the counter electrode. At the counter electrode, a reduction of triiodide in turn will regenerate iodide through donation of electrons from the external circuit to complete the circuit.

To fabricate the polymer matrix electrolyte for the DSSCs, we chose to introduce ethoxy silane moieties onto PB via the addition of thiols to double bonds, using MPTS as a reactant, as shown in Figure 1. By introducing ethoxy silane moieties onto PB, the PBS-Si could easily perform a hydrolysis-crosslinking reaction. The PBS-Si was analyzed by ^1^H NMR and compared with the initial PB spectrum. The spectrum of PBS-Si clearly showed the appearance of a triplet at 1.23 ppm and a quadruplet at 3.84 ppm assigned to the ethoxy groups.

**Figure 3:**
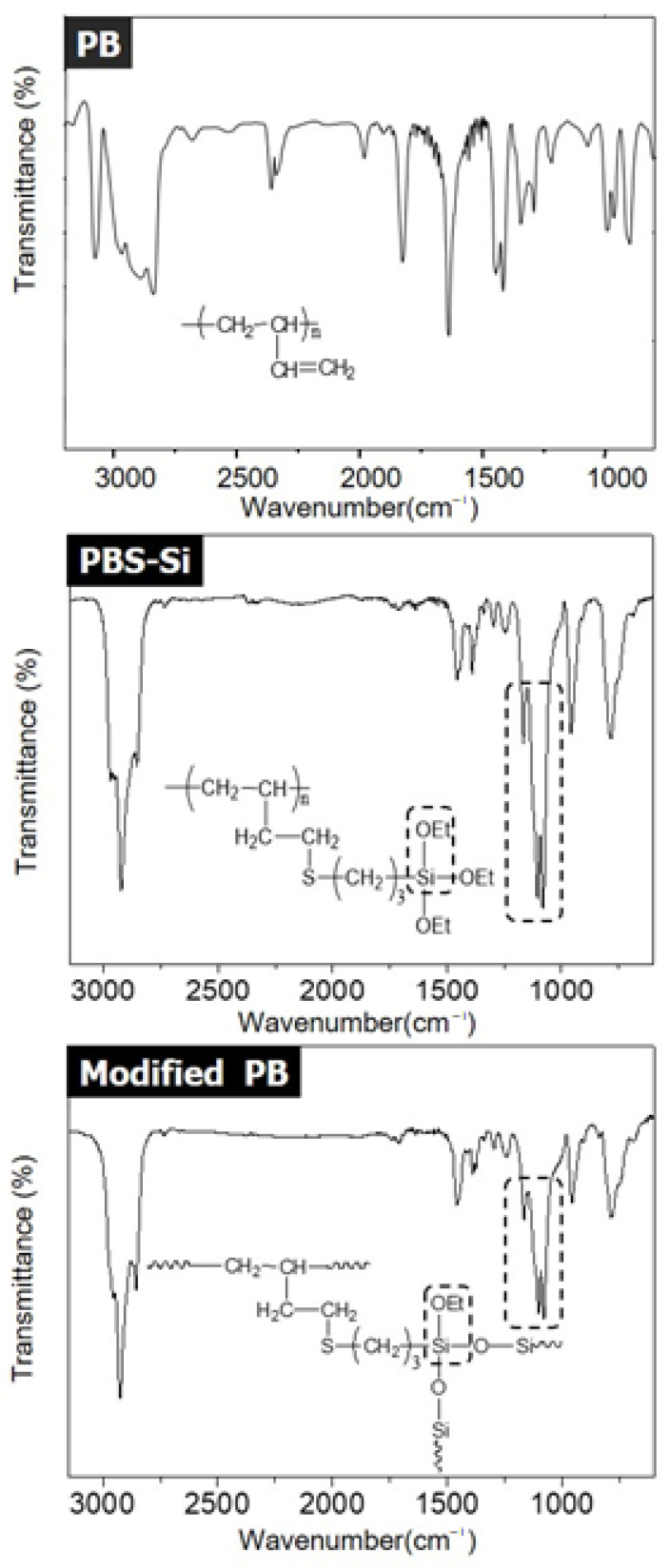
FT-IR spectra of PB, PBS-Si, and modified PB.

In Figure 3, the FT-IR spectra of PB, PBS-Si, and modified PB showed characteristic absorption peaks at 2975–2844 cm^−1^ due to CH stretching of the alkyl group, with peaks at 1169–1111 cm^−1^ and 956 cm^−1^ due to Si-O-C asym and sym stretching of the PBS-Si and modified PB, respectively. After gelation, the intensity at 956 cm^−1^ of Si-O-C sym stretching relatively decreased. It was found that Si-O-C groups gradually changed to Si-O-Si groups in polymer chains.

**Figure 4:**
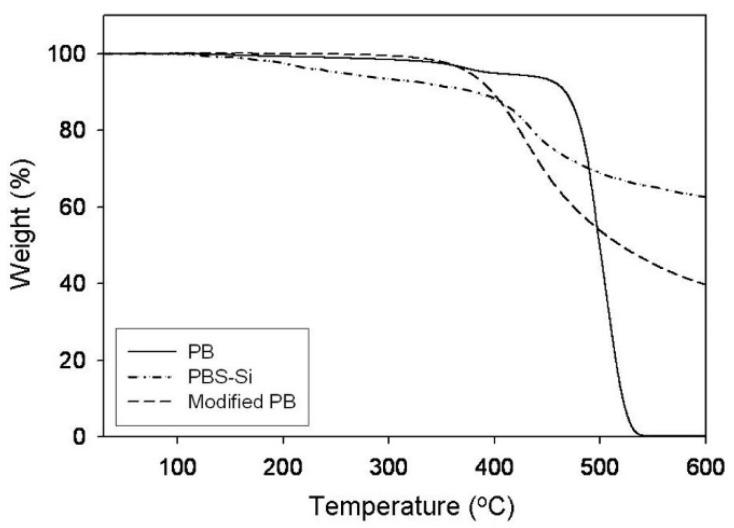
TGA curves of PB, PBS-Si, and modified PB.

The thermal properties of modified PB and unmodified PB at the initial decomposition temperature (T_d_) are listed in Table 1. The T_d_ values of PB, PBS-Si, and modified PB were observed at 497.54, 435.19, and 399.58 °C, respectively. As shown in Figure 4, the relatively large residue weights of PBS-Si and modified PB were attributed to the formation of a silica network structure in the main chain with the increase of temperature.

**Figure 5:**
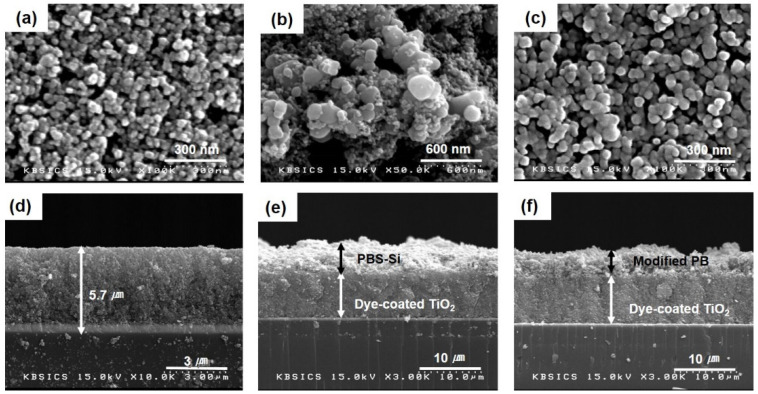
FESEM images of a dye-coated TiO_2_ film: (**a**,**d**) top and cross-section images before coating of PBS-Si; (**b**,**e**) top and cross-section images after coating of PBS-Si; (**c**,**f**) top and cross-section images after fabrication of Modified PB.

Figure 5a,d show top and cross-sectional FESEM images of the dye-coated TiO_2_ film before the coating of PBS-Si. Figure 5b,e show the corresponding images after the coating of PBS-Si. Figure 5c,f show the corresponding images of modified PB fabricated after 72 h of PBS-Si coating. Before the coating of PBS-Si, TiO_2_ nanoparticles were distinguishable with sizes of 10–30 nm. After the coating of PBS-Si, they were irregularly covered with the PBS-Si materials. The cross-section was also very rough film. In the modified PB, fabricated after 72 h of PBS-Si coating, the TiO_2_ particle sizes apparently increased, and the images of the nanoparticles became indistinct. These images clearly indicated that the modified PB penetrated deep into the pores of the TiO_2_ film and became uniformly distributed in the film. The thickness of the cross-section was a little shrunk compared to Figure 5e. The penetration and impregnation of modified PB, that resulted from the gelation, were essential for interfacial contact between the dye-coated TiO_2_ film and the electrolyte.

**Figure 6:**
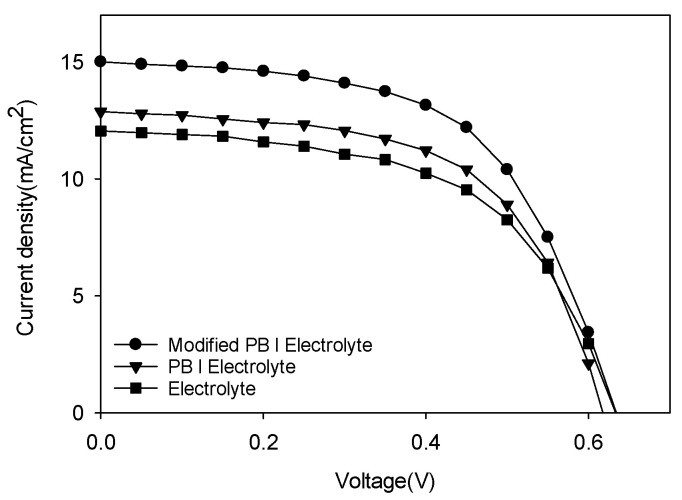
J-V curves of DSSC devices using modified PB, PB, and electrolyte at 72 h with AM 1.5.

The best I-V characteristics of DSSC devices using three kinds of electrolytes are summarized in Table 2. The fill factor can be calculated with Equation (1):

FF = P_max_ / (I_sc_ ∗ V_oc_) (1)

where P_max_ is the maximum electrical power obtained; I_sc_ and V_oc_ are the short-circuit current and the open-circuit voltage, respectively. The power conversion efficiency of the DSSC is obtained with Equation (2):

ƞ = P_max_ / (I ∗ A) = (I_sc_ ∗ V_oc_ ∗ FF) / (I ∗ A) (2)

where I is the intensity of incident light, and A is the cell area that is illuminated. Measurement of the device was performed at 72 h when the polymer matrix was sufficiently gelled to serve as a matrix. According to the J-V curves of FTO/TiO_2_/Dye/Polymer matrix electrolyte/Pt devices using modified PB or PB as a polymer matrix shown in Figure 6 and Table 2, their power conversion efficiencies were measured to be 5.49% and 4.68%, respectively. Photovoltaic parameters of twelve DSSC devices using Electrolyte, PB/Electrolyte, and Modified PB/Electrolyte within error ranges with average values and best efficiency results after 72 h under 100 Mw/cm^2^ at AM 1.5 are shown in Figure S6. In Table S1, photovoltaic data of V_oc_, J_sc_, FF, and Efficiency parameters of twelve DSSC devices using Electrolyte, PB/Electrolyte, and Modified PB/Electrolyte after 72 h under 100 Mw/cm^2^ at AM 1.5 are summarized. Best efficiency results showed at Dev1-2 of Electrolyte, Dev2-2 of PB/Electrolyte, and Dev1-2 of Modified PB/Electrolyte in Table S1. For comparison of results, the J-V data of FTO/TiO_2_/Dye/electrolyte/Pt devices using the electrolyte showed a lower efficiency of 4.29% under the same condition. While the modified PB layer did not show any improvement of V_oc_ or FF, J_sc_ was improved by the introduction of the layer effectively, giving a high conversion efficiency.

From these results, we found that the chemical structure of the polymer matrix in the quasi-solid electrolytes was affected by the photovoltaic performances of the DSSC devices. The lone pair electrons of the oxygen in the siloxane group of modified PB could capture the cations in the quasi-solid electrolytes. By the separation of cations in the quasi-solid electrolytes, redox couples (I^−^/I_3_^−^) were quickly formed and stabilized in the quasi-solid electrolyte. The stabilization of the redox couples (I^−^/I_3_^−^) in the quasi-solid electrolytes resulted in ionic transport, and the current flow increased. Therefore, the J_sc_ of the DSSC devices using modified PB was enhanced, and led to the improvement of efficiency.

**Figure 7:**
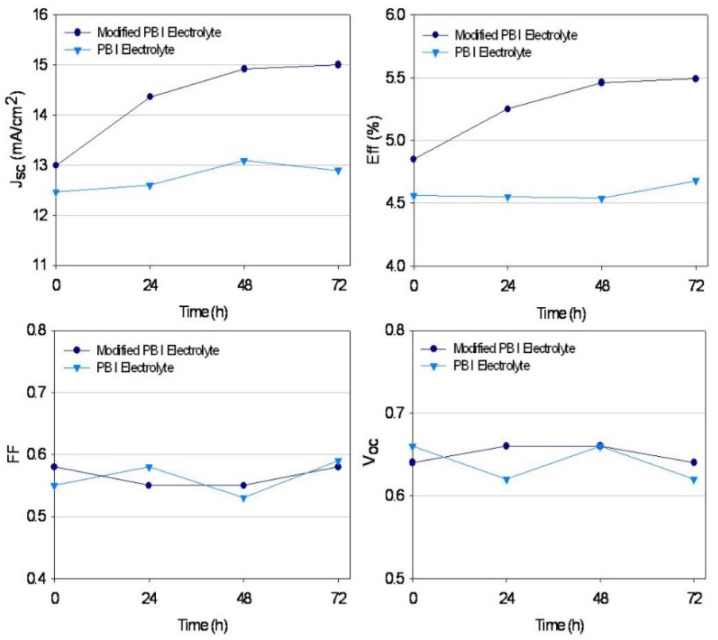
A plot showing values of short-circuit current density, open-circuit voltage, fill factor, and efficiency of DSSC device measured during 72 h.

Figure 7 presents variations in the photovoltaic parameters of the devices measured during 72 h. While the DSSC device with the modified PB did not show any improvement of V_oc_ or fill factor, the J_sc_ and efficiency of the device with the modified PB increased as the day progressed. With the modified PB, as shown in Figure 7, the initial values of J_sc_ and efficiency increased gradually and became stable after about 72 h, indicating that the gelation reaction continued inside the polymer. There was little change in all parameters of the DSSC device with PB after 72 h, because no gelation reaction occurred in PB. These results demonstrated that the modified PB carried out the cross-linking reaction by itself. The -O-Si-O- bonds by cross-linking reaction offer better interfacial contact of the electrolyte.

The conductivity of the polymer matrix electrolyte was measured using electrochemical impedance spectroscopy (EIS). Conductivity measurements were made by placing the polymer layer and model electrolyte between two transparent conductive oxide (TCO) slides. The conductivity (*σ*) was given by Equation (3):

(3) $$\sigma =\frac{1}{R}\times \frac{\ell }{A}$$

where *R* was the resistance, *ℓ* was the length or thickness, and *A* was the active area. The conductivity results are shown in Table 3. The conductivity of the polymer matrix electrolyte based on modified PB reached 3.39 × 10^−3^ S/cm. This result was attributed to the better interfacial contact and stability of the modified PB.

**Figure 8:**
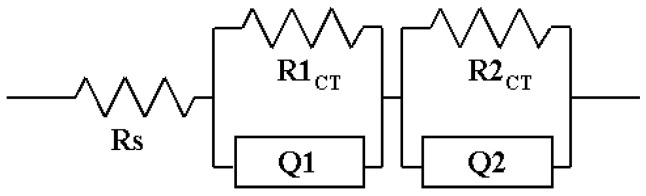
An equivalent circuit for EIS for DSSCs based on modified PB, PB, and electrolyte; Rs: Series resistance; R1_CT_: charge transfer resistance of Pt/electrolyte interface; R2_CT_: charge transfer resistance of TiO_2_/Dye/electrolyte interface; Q1 and Q2: constant phase element.

To confirm the effect of the interfacial charge transfer on the photovoltaic performance of the DSSC device, the interfacial charge transfer resistance was investigated by EIS measurement. The Nyquist plots of the DSSCs based on the modified PB/electrolyte, PB/electrolyte, and electrolyte in dark during 72 h are shown in Figures S3–S5, respectively. The charge transfer resistances of the FTO/TiO_2_/Dye/Electrolyte/Pt cells according to measured time are listed in Table 4. R_S_, R1_CT_, and R2_CT_ are series resistance, charge transfer resistance of the Pt/electrolyte interface, and charge transfer resistance of the TiO_2_/Dye/electrolyte interface, respectively. R _CT_s were obtained by fitting with Z-MAN software. The results of R_CT_s are summarized in Table 4 and the equivalent circuit is shown in Figure 8. Almost all DSSC devices had similar R_S_ values of about 22~26 Ω. After 72 h, the R_S_ did not change significantly. The DSSC devices using modified PB showed lower R1_CT_ values (9.7~11.6 Ω) than other devices (PB: 18.6~33.7 Ω; electrolyte: 17.2~38.1 Ω). This device is considered to have good charge transfer between the Pt electrode and the modified PB electrolyte. These results are considered to cause an increase in current density. The R2_CT_ values of the DSSC devices did not show as significant a difference as the change of electrolyte. When using an electrolyte, the resistance increases rapidly due to leakage of the electrolyte over time compared to the initial value. However, in the case of the modified PB, there was no significant change compared to other electrolytes.

To pursue commercial production and scale up the manufacturing process by replacing traditional liquid electrolytes presents problems such as reliability, less long-term stability, and the need for hermetic sealing; the long-term stability of the DSSC system has been a subject of concern throughout the years of technology development. The overall stability of the DSSC is controlled by two factors, namely physical stability and chemical stability. Physical stability is related to possible evaporation of the liquid electrolyte at elevated temperatures. This is a technological problem, for which solutions include the use of suitable sealing materials and techniques [36,37]. The intrinsic chemical stability is related to irreversible electrochemical and thermal degradation of the dye or electrolyte components, which might occur during the operation of the DSSC.

**Figure 9:**
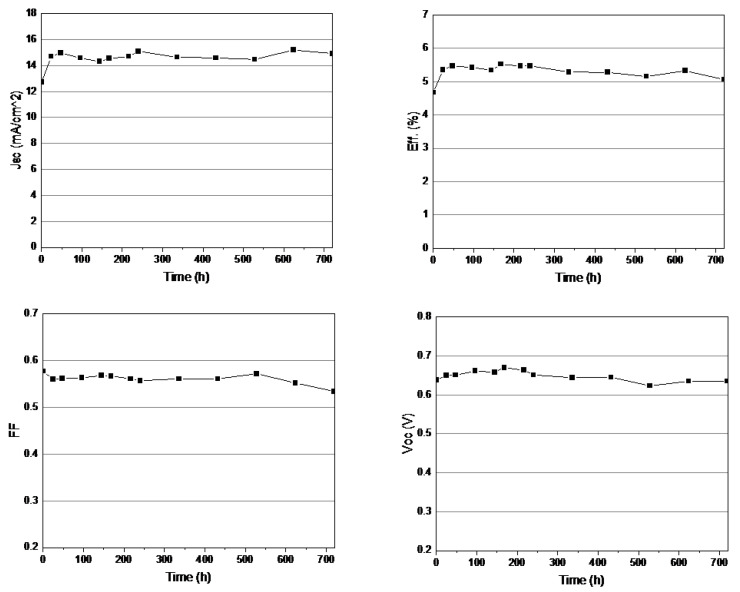
Temporal evolution of photovoltaic parameters of DSSC devices using modified PB during storage in darkness at room temperature for 720 h.

In particular, operational stability is one of the most important factors affecting a photovoltaic device. This has been overcome, to a large extent, by using good sealing methods and appropriate chemicals. In the present study, DSSC devices using modified PB were fabricated for long-term stability tests. DSSC devices were stored in darkness at room temperature for 720 h, and their photovoltaic performance stabilities were measured using a Solar Simulator. Figure 9 shows the detailed evaluation results of the device parameters during 720 h of aging at room temperature in air. As shown in Figure 9, the J_sc_ and efficiency were drastically increased during the initial gelation. The DSSC device also maintained over 90% of its initial V_oc_ and FF after 720 h (V_oc_ = 0.635 V, J_sc_ = 14.93 mA/cm^2^, FF = 0.53, Eff. = 5.06% after 720 h). The photovoltaic parameters showed little change during 720 h of aging after the gelation. These very encouraging results will foster practical applications of DSSCs. This result indicates that modified PB in DSSC devices might have exceeded chemical reactions by the sol-gel process during aging in long-term stability tests. No more increase of efficiency was found after the gelation was over.

In order to comparing the long-term stability of DSSC devices as the electrolyte, DSSC devices using PB and electrolyte were also fabricated and tested in the same conditions. Due to sealants, the efficiency of DSSC devices using PB was maintained at about 52% until 96 h (J_sc_ = 8.8 mA/cm^2^, Eff. = 2.45%). However, this device dramatically decreased after 144 h; eventually, this device showed no current and 0% of efficiency after seven days. In DSSC devices using electrolyte, the sealant was gradually corroded with the liquid-type electrolyte, and most electrolytes were evaporated through the damaged sealant after five days. Eventually, the DSSC device using electrolyte showed no current and 0% of efficiency after five days.

From these results, we found that the siloxane group of modified PB affected the stabilization of the redox couples (I^−^/I_3_^−^) in the quasi-solid electrolyte. With the PB matrix, tetabutylammonium ions (cations) were dissociated from TBAI and the redox couples (I^−^/I_3_^−^) were easily generated in the quasi-solid electrolyte. Due to entanglement of the polymer chains, tetabutylammonium ions (cations) were able to remain inside the PB matrix initially. However, because the PB did not contain the siloxane group, the interaction of the tetabutylammonium ions (cations) with the PB matrix gradually weakened, and they escaped from the PB matrix. Therefore, the stability of the redox couples (I^−^/I_3_^−^) in the quasi-solid electrolyte using PB was reduced, and the long-term stability of the DSSC devices was also affected.

Recently, we have been searching for a DSSC with a lower cost and broader applicability using flexible electrodes. Flexible electrodes such as the use of films of polyethylene naphthalate coated with tin-doped indium oxide (ITO-PEN) show lower cost, with some technological advantages considering aspects such as fragility as well as form and the shape limitations of glass electrodes. A flexible DSSC was assembled using flexible electrodes, a modified PB electrolyte, and a Pt-coated counter electrode. ITO-PEN (thickness, 200 um; sheet resistance, 13 Ω sq^−1^; transmittance, 80%, Peccell Technologies) was coated with a 5 um-thick nanoporous TiO_2_ layer by doctor-blading of a binder-free nanocrystalline TiO_2_ paste, as reported previously [38]. The J-V characteristics of the flexible DSSC device are summarized in Table 5. The current density and efficiency for the flexible version of the DSSC were approximately half of those observed for the cell assembled with glass-FTO electrodes. The main reason for the lower performance of the flexible cell must have been the poor electronic contact between the TiO_2_ nanoparticles and the ITO-PEN plastic substrates. However, these results have plenty of potential for improvement, to develop more advanced DSSCs.

## 4. Conclusions

The modified PB was successfully synthesized by a sol-gel process. We constructed a DSSC device using the modified PB as a polymer matrix in electrolyte. A DSSC based on the modified PB matrix electrolyte showed a power conversion efficiency of 5.49% at AM 1.5 and a light intensity of 100 mW/cm^2^. In DSSCs using the modified PB matrix electrolyte, the current density and the power conversion efficiency increased dramatically during 72 h of gelation, showing excellent durability while maintaining 90% of initial value after 720 h. This study demonstrates that using a modified PB matrix electrolyte is one of methods to increase photovoltaic performances and improve the stability of DSSC devices, due to the high conductivity and good interfacial charge transfer of the modified PB matrix electrolyte.

Our study presents a successful method for modifying a linear polymer without a gelling agent, and confirms the application potential of new modified polymers as an electrolyte. The development of new modified polymers can further improve the properties and performances of quasi-solid electrolytes, so as to replace liquid electrolytes, and lead to the excellent long-term stability and commercial application of DSSCs. Furthermore, for commercialization of DSSCs, polymer-based electrolytes provide the required mechanical strength and flexibility to endure bending stress, which is quite advantageous for both the roll-to-roll process and flexible devices.

## Figures and Tables

**Table 1:** Initial Thermal Decomposition Temperatures (T_d_’s) of PB, PBS-Si, and modified PB.

	T_d,5%_ (°C) ^1^	T_d_ (°C) ^1^
Modified PB	345.66	399.58
PBS-Si	254.38	435.19
PB	395.81	497.54

^1^ TGA measurement on the second heating, at a heating rate of 10 °C/min (nitrogen atmosphere).

**Table 2:** J-V characteristic data of DSSC devices using modified PB, PB, and electrolyte after 72 h at AM 1.5.

	V_oc_ (V)	J_sc_ (mA/cm^2^)	FF	Eff. (%)
Modified PB/Electrolyte	0.64	15.00	0.58	5.49
PB/Electrolyte	0.62	12.88	0.59	4.68
Electrolyte	0.64	12.05	0.56	4.29

**Table 3:** The conductivity of electrolyte of modified PB, PB, and electrolyte by EIS.

	Conductivity (S/cm)
Modified PB/Electrolyte	3.39 × 10^−3^
PB/Electrolyte	2.73 × 10^−3^
Electrolyte	2.20 × 10^−3^

**Table 4:** EIS data for DSSCs based on modified PB, PB, and electrolyte in darkness for 72 h.

	Measured Time (h)	R_S_^1^ (Ω)	R1_CT_^2^ (Ω)	R2_CT_^3^ (Ω)
Modified PB/Electrolyte	0	24.8	9.7	15.1
	24	26.0	10.7	26.4
	48	24.2	11.6	28.2
	72	24.9	11.4	31.0
PB/Electrolyte	0	24.1	18.6	9.0
	24	25.6	23.8	21.3
	48	25.1	33.7	28.7
	72	24.6	33.1	29.3
Electrolyte	0	23.5	17.2	9.5
	24	24.6	62.1	17.9
	48	25.9	35.8	26.8
	72	22.8	38.1	27.4

^1^ The series resistance of the DSSC device; ^2^ the charge transfer resistance of the Pt/electrolyte interface; ^3^ the charge transfer resistance of the TiO_2_/Dye/electrolyte interface.

**Table 5:** J-V characteristic data of a flexible DSSC device using an ITO-PEN plastic substrate and using modified PB after 72 at AM 1.5.

	V_oc_ (V)	J_sc_ (mA/cm^2^)	FF	Eff. (%)
Modified PB/Electrolyte	0.70	6.30	0.62	2.77

## Data Availability

Not applicable.

## Supplementary Materials

The following supporting information can be downloaded at: https://www.mdpi.com/article/10.3390/polym14122347/s1, Figure S1: ^1^H NMR (300 MHz, CDCl_3_) of 1,2-addition polybutadiene (PB); Figure S2: ^1^H NMR (300 MHz, CDCl_3_) of PBS-Si; Figure S3: Nyquist plots of DSSCs based on modified PB/electrolyte in darkness for 72 h (**a**) 0 h, (**b**) 24 h, (**c**) 48 h, (**d**) 72 h by EIS; Figure S4: Nyquist plots of DSSCs based on PB/electrolyte in darkness for 72 h (**a**) 0 h, (**b**) 24 h, (**c**) 48 h, (**d**) 72 h by EIS; Figure S5: Nyquist plots of DSSCs based on electrolyte in darkness for 72 h (**a**) 0 h, (**b**) 24 h, (**c**) 48 h, (**d**) 72 h by EIS; Figure S6: Photovoltaic parameters of twelve DSSC devices (Dev1-1~Dev3-4) using Electrolyte (blue), PB/Electrolyte (orange), and Modified PB/Electrolyte (gray) within error ranges with average values and best efficiency results after 72 h under 100 Mw/cm^2^ at AM 1.5; Table S1: Photovoltaic data of V_oc_, J_sc_, FF, and Efficiency parameters of twelve DSSC devices (Dev1-1~Dev3-4) using Electrolyte, PB/Electrolyte, and Modified PB/Electrolyte after 72 h under 100 Mw/cm^2^ at AM 1.5. Best efficiency results showed at Dev1-2 of Electrolyte, Dev2-2 of PB/Electrolyte, and Dev1-2 of Modified PB/Electrolyte.

## References

[1] O’Regan B., Gratzel M. (1991). A low-cost, high-efficiency solar cell based on dye-sensitized colloidal TiO_2_ films. Nature.

[2] Sharma K., Sharma V., Sharma S.S. (2018). Dye-Sensitized Solar Cells: Fundamentals and Current Status. Nanoscale Res. Lett..

[3] Nazeeruddin K., Baranoff E., Gratzel M. (2011). Dye-sensitized solar cells: A brief overview. Sol. Energy.

[4] Altobello S., Bignozzi C.A., Caramori S., Larramona G., Quici S., Marzanni G., Lakhmiri R. (2004). Sensitization of TiO_2_ with ruthenium complexes containing boronic acid functions. J. Photochem. Photobiol. A Chem..

[5] Kunzmann A., Valero S.E., Sepúlveda A., Rico-Santacruz M., Lalinde E.R., Berenguer J., García-Martínez J.M., Guldi D., Serrano E.D., Costa R. (2018). Hybrid Dye-Titania Nanoparticles for Superior Low-Temperature Dye-Sensitized Solar Cells. Adv. Energy Mat..

[6] Yu X., Li Z., Sun X., Zhong C., Zhu Z., Li Z., Jen A.K.-Y. (2021). Dopant-free dicyanofluoranthene-based hole transporting material with low cost enables efficient flexible perovskite solar cells. Nano Energy.

[7] Kyeong M., Lee J., Lee K., Hong S. (2018). BODIPY-Based Conjugated Polymers for Use as Dopant-Free Hole Transporting Materials for Durable Perovskite Solar Cells: Selective Tuning of HOMO/LUMO Levels. ACS Appl. Mater. Interfaces.

[8] Smestad G.P., Spiekermann S., Kowalik J., Grant C.D., Schwartzberg A.M., Zhang J., Tolbert L.M., Moons E. (2003). A technique to compare polythiophene solid-state dye sensitized TiO_2_ solar cells to liquid junction devices. Sol. Energy Mater. Sol. Cells.

[9] Fukuri N., Masaki N., Kitamura T., Wada Y., Yanagida S. (2006). Electron Transport Analysis for Improvement of Solid-State Dye-Sensitized Solar Cells Using Poly(3,4-ethylenedioxythiophene) as Hole Conductors. J. Phys. Chem. B.

[10] Krueger J., Plass R., Graetzel M., Matthieu H. (2002). Improvement of the photovoltaic performance of solid-state dye-sensitized device by silver complexation of the sensitizer cis-bis(4,4′cis-bis(4,4′-dicarboxy-2,2′bipyridine)-bis(isothiocyanato)2,2′bipyridine)-bis(isothiocyanato) ruthenium(II). J. Appl. Phys. Lett..

[11] Cao Y., Li Y., Morrissey T., Lam B., Patrick B.O., Dvorak D.J., Xia Z., Kelly T.L., Berlinguette C.P. (2019). Dopant-free molecular hole transport material that mediates a 20% power conversion efficiency in a perovskite solar cell. Energy Environ. Sci..

[12] O’Regan B., Lenzmann F., Muis R., Wienke J. (2002). A Solid-State Dye-Sensitized Solar Cell Fabricated with Pressure-Treated P25−TiO_2_ and CuSCN:  Analysis of Pore Filling and IV Characteristics. Chem. Mater..

[13] O’Regan B., Schwartz D.T., Zakeeruddin X.M., Graetzel M. (2000). Electrodeposited Nanocomposite n–p Heterojunctions for Solid-State Dye-Sensitized Photovoltaics. Adv. Mater..

[14] Meng Q.B., Takahashi K., Zhang X.T., Sutanto I., Rao T.N., Sato O., Fujishima A. (2003). Fabrication of an Efficient Solid-State Dye-Sensitized Solar Cell. Langmuir.

[15] Mahrov B., Boschloo G., Hagfeldt A., Siegbahn H., Rensmo H. (2004). Photoelectron Spectroscopy Studies of Ru(dcbpyH_2_)_2_(NCS)_2_/CuI and Ru(dcbpyH_2_)_2_(NCS)_2_/CuSCN Interfaces for Solar Cell Applications. J. Phys. Chem. B.

[16] Nogueira A.F., Longo C., De Paoli M.A. (2004). Polymers in dye sensitized solar cells: Overview and perspectives. Coord. Chem. Rev..

[17] Stergiopoulos T., Arabatzis I.M., Katsaros G., Falaras P. (2002). Binary Polyethylene Oxide/Titania Solid-State Redox Electrolyte for Highly Efficient Nanocrystalline TiO_2_ Photoelectrochemical Cells. Nano Lett..

[18] Wang P., Zakeeruddin S.M., Humphry-Baker R., Moser J.E., Graetzel M. (2003). A stable quasi-solid-state dye-sensitized solar cell with an amphiphilic ruthenium sensitizer and polymer gel electrolyte. Adv. Mater..

[19] Asano T., Kubo T., Nishikitani Y. (2004). Electrochemical properties of dye-sensitized solar cells fabricated with PVDF-type polymeric solid electrolytes. J. Photochem. Photobiol. A.

[20] Komiya R., Han L., Yamanaka R., Islam A., Mitate T. (2004). Highly efficient quasi-solid state dye-sensitized solar cell with ion conducting polymer electrolyte. J. Photochem. Photobiol. A.

[21] Hagfeldt A., Boschloo G., Sun L., Kloo L., Pettersson H. (2010). Dye-Sensitized Solar Cells. Chem. Rev..

[22] Wu J., Lan Z., Hao S., Li P., Lin J., Huang M., Fang L., Huang Y. (2008). Progress on the electrolytes for dye-sensitized solar cells. Pure Appl. Chem..

[23] Gorlov M., Kloo L. (2008). Ionic liquid electrolytes for dye-sensitized solar cells. Dalton Trans..

[24] Zakeeruddin S., Gratzel M. (2009). Solvent-Free Ionic Liquid Electrolytes for Mesoscopic Dye-Sensitized Solar Cells. Adv. Funct. Mater..

[25] Marino I.G., Bersani D., Lottici P.P. (2001). Holographic gratings in DR1-doped sol–gel silica and ORMOSILs thin films. Opt. Mat..

[26] Riehl D., Chaput F., Lévy Y., Boilot J.P., Kajzar F., Chollet P.A. (1995). Second-order optical nonlinearities of azo chromophores covalently attached to a sol-gel matrix. Chem. Phys. Lett..

[27] Novak B.M. (1993). Hybrid Nanocomposite Materials—Between inorganic glasses and organic polymers. Adv. Mater..

[28] Hopfield J.J., Onuchic J.N., Beratan D.N. (1988). A Molecular Shift Register Based on Electron Transfer. Science.

[29] Song D., Cho W., Lee J., Kang Y. (2014). Toward Higher Energy Conversion Efficiency for Solid Polymer Electrolyte Dye-Sensitized Solar Cells: Ionic Conductivity and TiO_2_ Pore-Filling. J. Phys. Chem. Lett..

[30] Noto V.D., Lavina S., Giffin G.A., Negro E., Scrosati B. (2011). Polymer electrolytes: Present, past and future. Electrochim. Acta.

[31] Kang M., Ahn K., Lee J. (2008). Quasi-solid-state dye-sensitized solar cells employing ternary component polymer-gel electrolytes. J. Power Sources.

[32] Schapman F., Couvercelle J.P., Bunel C. (1998). Low molar mass polybutadiene made crosslinkable by the introduction of silane moities via urethane linkage: 1. Synthesis and kinetic study. Polymers.

[33] Schapman F., Couvercelle J.P., Bunel C. (1998). Low molar mass polybutadiene made crosslinkable by the introduction of silane moieties via urethane linkage: 2. Crosslinking study. Polymers.

[34] Lee H.J., Lee J.K., Kim M.R., Shin W.S., Jin S.H., Park S.W., Kim K.H., Park D.W., Park S.W. (2007). Influence of Ionic Liquids in Quasi-Solid State Electrolyte on Dye-Sensitized Solar Cell Performance. Mol. Cryst. Liq. Cryst..

[35] Lee J.K., Kim W.S., Lee H.J., Shin W.S., Jin S.H., Lee W.K., Kim M.R. (2006). Preparations and Photovoltaic Properties of Dye-sensitized Solar Cells Using Thiophene-based Copolymers as Polymer Electrolytes. Polym. Adv. Technol..

[36] Hinsch A., Wolf M. (1996). Method of Manufacturing a Module of Photo-Electrochemical Cells with Long Term Efficiency. Patent.

[37] Kurth M. (2000). Solar Module. Patent.

[38] Kijitori Y., Ikegami M., Miyasaka T. (2007). Highly Efficient Plastic Dye-sensitized Photoelectrodes Prepared by Low-temperature Binder-free Coating of Mesoscopic Titania Pastes. Chem. Lett..

