# Supplementary material for: Photovoltaic Performances of Dye-Sensitized Solar Cells Based on Modified Polybutadiene Matrix Electrolytes by Sol-Gel Process
Source: Polymers (Basel). 2022 Jun 9;14(12):2347. doi: 10.3390/polym14122347 (PMC9229922; doi:10.3390/polym14122347)
Supplement: Supplementary file 1 [file polymers-14-02347-s001.zip › polymers-1750324-supplementary.pdf]

Supporting information for "Photovoltaic Performances of Dye-Sensitized Solar Cells Based on Modified Polybutadiene Matrix Electrolytes by Sol-Gel Process"

Mi-Ra Kim [1], Thanh Chung Pham [3], Hyun-Seock Yang [4], Sung Heum Park [4] and Songyi Lee [1,2,*]

1 Department of Chemistry, Pukyong National University, Busan 48513, Korea; mrkim2@pknu.ac.kr
2 Industry 4.0 Convergence Bionics Engineering, Pukyong National University, Busan 48513, Korea
3 Division of Chemical Engineering and Materials Science, Ewha Womans University, Seoul 03760, Korea; ptchung.chem@gmail.com
4 Department of Physics, Pukyong National University, Busan 48513, Korea; randafine@gmail.com (H.-S.Y.); spark@pknu.ac.kr (S.H.P.)
* Correspondence: slee@pknu.ac.kr

Contents

1. NMR spectra data........ Page S2
2. EIS results........ Page S3-5
3. Photovolatic parameter results........ Page S6
4. Photovolatic data........ Table S1

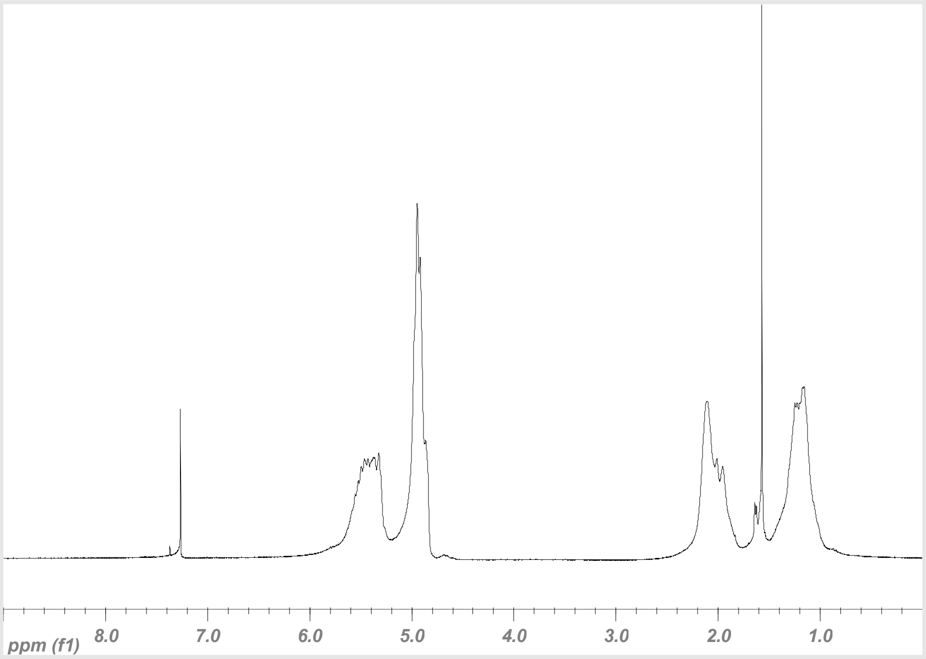

Figure S1. ^1H NMR (300 MHz, $CDCl_3$) of 1,2-addition polybutadiene (PB).

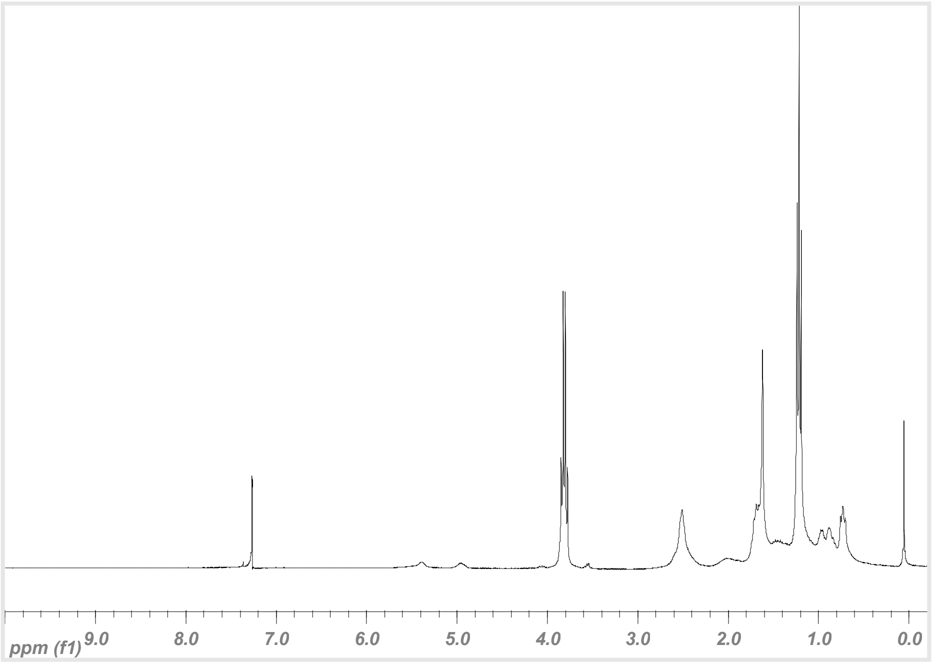

Figure S2. ^1H NMR (300 MHz, $CDCl_3$) of PBS-Si.

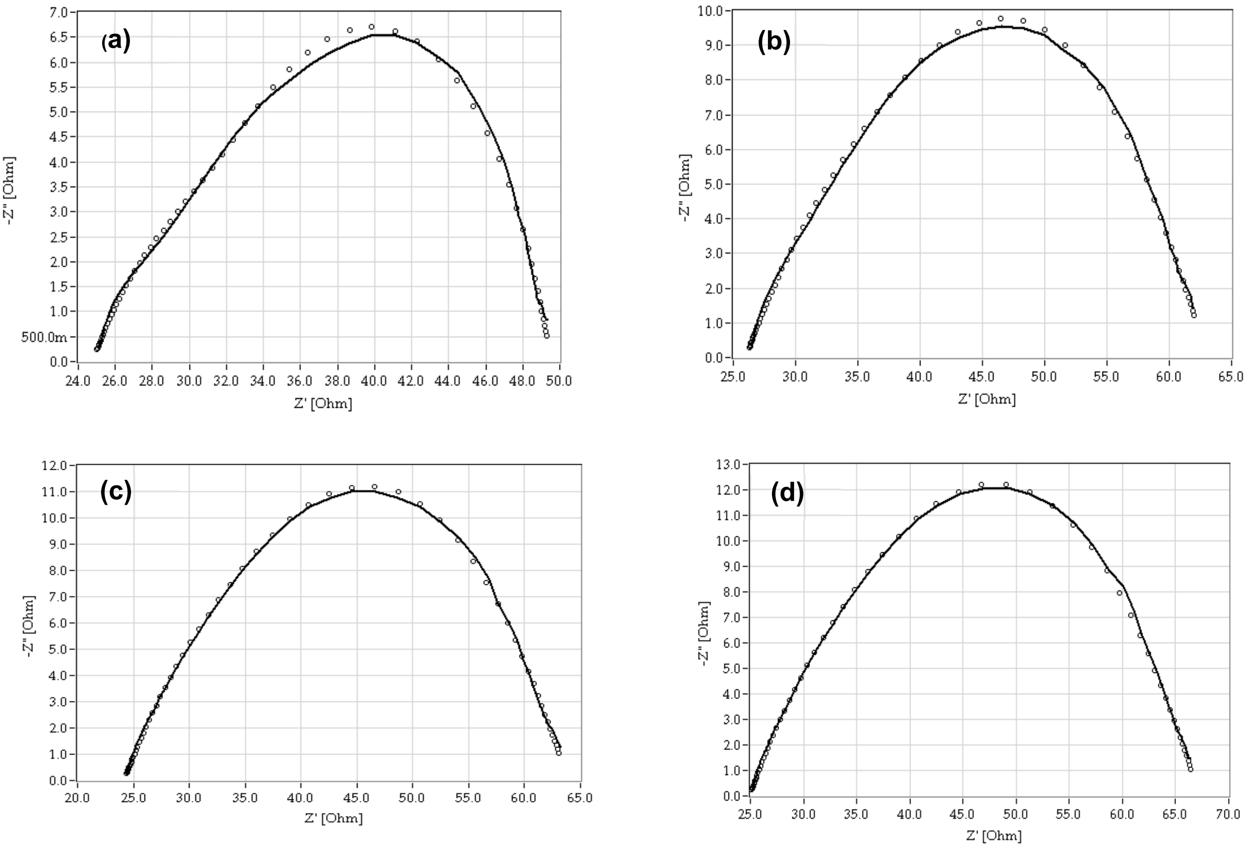

Figure S3. Nyquist plots of DSSCs based on the Modified PB / Electrolyte in dark during 72 hours (a) 0h, (b) 24h, (c) 48h, (d) 72h by EIS.

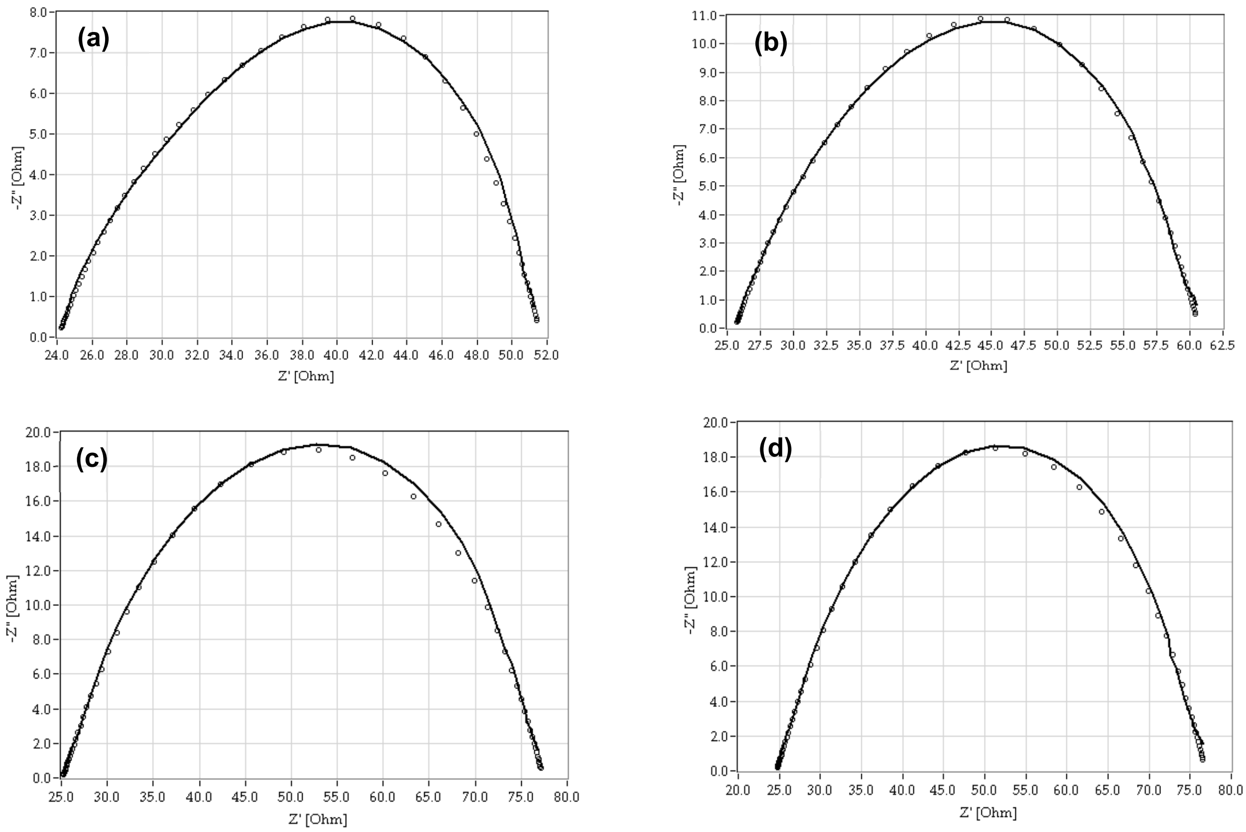

Figure S4. Nyquist plots of DSSCs based on PB / Electrolyte in dark during 72 hours (a) 0h, (b) 24h, (c) 48h, (d) 72h by EIS.

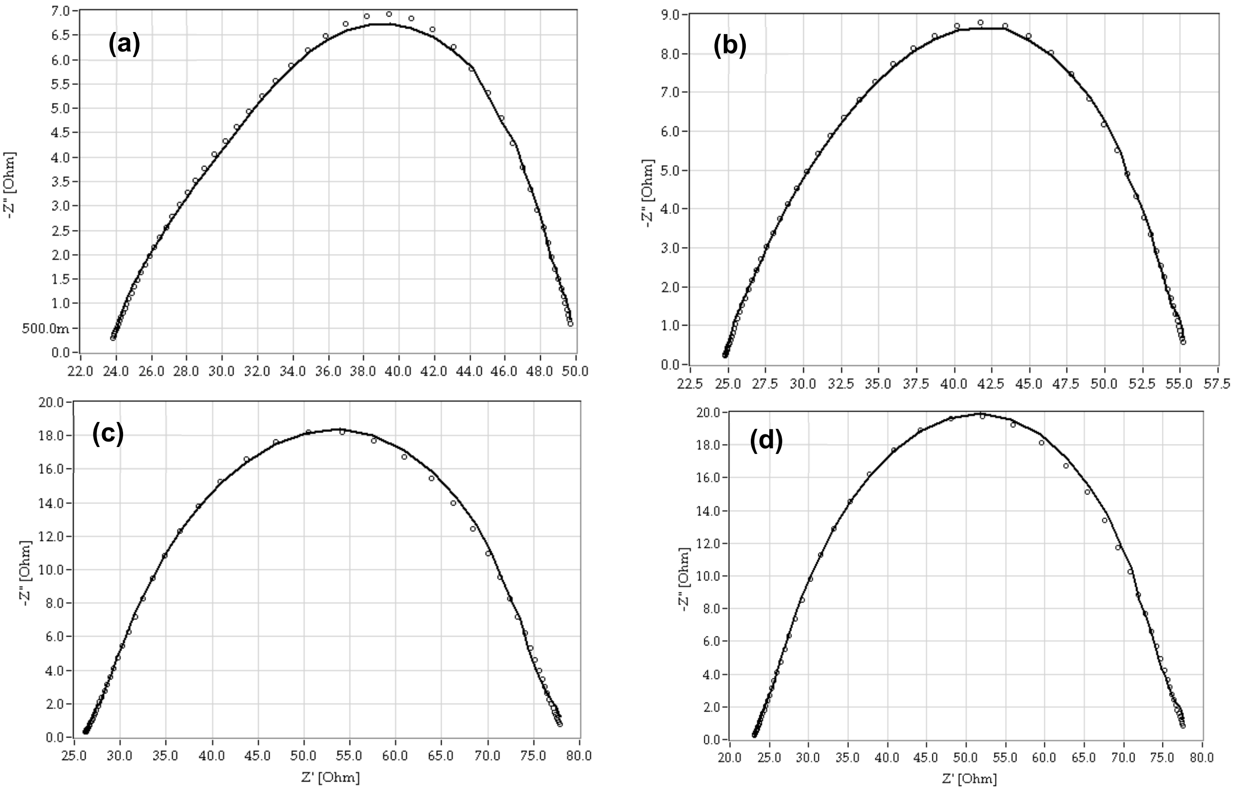

Figure S5. Nyquist plots of DSSCs based on Electrolyte in dark during 72 hours (a) 0h, (b) 24h, (c) 48h, (d) 72h by EIS.

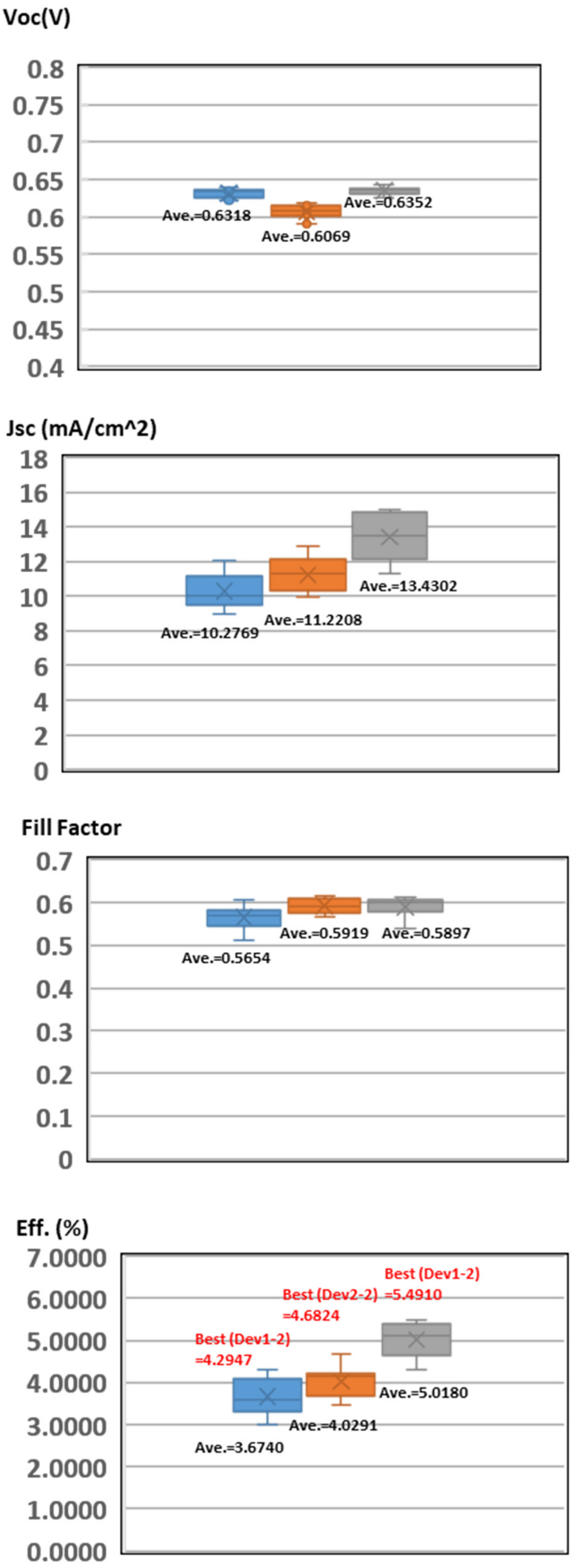

Figure S6. Photovoltaic parameters of twelve DSSC devices (Dev1-1~Dev3-4) using Electrolyte (blue), PB/Electrolyte (orange), and Modified PB/Electrolyte (gray) within error ranges with average values and best efficiency results after 72 h under 100 Mw/cm^2 at AM 1.5.

Table S1. Photovoltaic data of V_{oc}, J_{sc}, FF, and Efficiency parameters of twelve DSSC devices (Dev1-1~Dev3-4) using Electrolyte, PB/Electrolyte, and Modified PB/Electrolyte after 72 h under 100 Mw/cm^2 at AM 1.5. Best efficiency results showed at Dev1-2 of Electrolyte, Dev2-2 of PB/Electrolyte, and Dev1-2 of Modified PB/Electrolyte.

		Electrolyte	PB/Electrolyte	Modified PB/Electrolyte
V_{oc}(V)	**Dev1-1**	0.6222	0.6123	0.6380
	**Dev1-2**	**0.6357**	0.6186	**0.6380**
	**Dev1-3**	0.624	0.6125	0.6368
	**Dev1-4**	0.6228	0.6181	0.6333
	**Dev2-1**	0.6392	0.6166	0.6304
	**Dev2-2**	0.6354	**0.6159**	0.6257
	**Dev2-3**	0.6307	0.6034	0.6304
	**Dev2-4**	0.6262	0.6029	0.6352
	**Dev3-1**	0.6369	0.5906	0.6404
	**Dev3-2**	0.6338	0.5966	0.6434
	**Dev3-3**	0.6393	0.5902	0.6332
	**Dev3-4**	0.6363	0.6056	0.6373
		**Electrolyte**	**PB/Electrolyte**	**Modified PB/Electrolyte**
Jsc (mA/cm^2)	**Dev1-1**	10.024	9.9520	13.8648
	**Dev1-2**	**12.049**	11.0268	**14.9551**
	**Dev1-3**	11.5608	11.6056	14.4812
	**Dev1-4**	11.4084	11.5868	14.494
	**Dev2-1**	9.104	11.5180	12.1612
	**Dev2-2**	9.0048	**12.8812**	12.1832
	**Dev2-3**	9.3232	10.364	11.3336
	**Dev2-4**	9.8964	10.3872	11.5440
	**Dev3-1**	9.8964	12.5080	14.9380
	**Dev3-2**	10.0572	12.3396	14.9780
	**Dev3-3**	10.5484	10.2064	13.0684
	**Dev3-4**	10.4504	10.2740	13.1612
		**Electrolyte**	**PB/Electrolyte**	**Modified PB/Electrolyte**
Fill Factor	**Dev1-1**	0.5721	0.5974	0.6001
	**Dev1-2**	**0.5607**	0.6013	0.5755
	**Dev1-3**	0.5756	0.5873	0.5868
	**Dev1-4**	0.5832	0.5899	0.5921
	**Dev2-1**	0.5689	0.6124	0.6095
	**Dev2-2**	0.5761	**0.5902**	0.6111
	**Dev2-3**	0.5112	0.6109	0.6023
	**Dev2-4**	0.5341	0.6158	0.6043
	**Dev3-1**	0.5671	0.5674	0.5401
	**Dev3-2**	0.6066	0.5728	0.5502
	**Dev3-3**	0.5906	0.5732	0.5997
	**Dev3-4**	0.5387	0.5847	0.6049
		**Electrolyte**	**PB/Electrolyte**	**Modified PB/Electrolyte**
Eff.(%)	**Dev1-1**	3.5681	3.6403	5.3083
	**Dev1-2**	**4.2947**	4.1016	**5.4910**
	**Dev1-3**	4.1523	4.1748	5.4113
	**Dev1-4**	4.1437	4.2247	5.4349
	**Dev2-1**	3.3106	4.3493	4.6727
	**Dev2-2**	3.2962	**4.6824**	4.6584
	**Dev2-3**	3.0059	3.8203	4.3033
	**Dev2-4**	3.3099	3.8564	4.4312
	**Dev3-1**	3.5744	4.1915	5.1668
	**Dev3-2**	3.8666	4.2168	5.3022
	**Dev3-3**	3.9828	3.4529	4.9625
	**Dev3-4**	3.5821	3.6380	5.0737
